# Carer preparedness improved by providing a supportive educational intervention for carers of patients with high-grade glioma: RCT results

**DOI:** 10.1007/s11060-023-04239-0

**Published:** 2023-01-19

**Authors:** Georgia K. B. Halkett, Elizabeth A. Lobb, Jane L. Phillips, Emma McDougall, Jenny Clarke, Rachel Campbell, Haryana M. Dhillon, Kevin McGeechan, Peter Hudson, Anne King, Helen Wheeler, Marina Kastelan, Anne Long, Anna K. Nowak, the Care-IS Project Team, Jade Newton, Laura Emery, Marie Gilbert, Robyn Atwood, Lisa Miller, Meera Agar, Rachael Moorin, Therese Shaw, Max Bulsara

**Affiliations:** 1 Curtin School of Nursing/Curtin Health Innovation Research Institute, Faculty of Health Sciences, Curtin University, GPO Box U1987, Bentley, Perth, WA 6005 Australia; 2 Calvary Health Care Kogarah, Sydney, NSW Australia; 3 School of Medicine, The University of Notre Dame, Sydney, NSW Australia; 4 Faculty of Health, University of Technology Sydney, Ultimo, NSW Australia; 5 School of Nursing, Faculty of Health, Queensland University of Technology, Brisbane, QLD Australia; 6 Psycho-Oncology Cooperative Research Group, School of Psychology, Faculty of Science, University of Sydney, Sydney, NSW Australia; 7 Centre for Medical Psychology & Evidence-Based Decision-Making, University of Sydney, Sydney, NSW Australia; 8 School of Public Health, University of Sydney, Sydney, NSW Australia; 9 Centre for Palliative Care St Vincent’s Hospital Melbourne, Fitzroy, VIC Australia; 10 Department of Nursing, University of Melbourne, Melbourne, VIC Australia; 11 Vrije University Brussels, Brussels, Belgium; 12 Cancer Network Western Australia, North Metropolitan Health Service, Perth, WA Australia; 13 Northern Sydney Cancer Centre, Royal North Shore Hospital, St Leonards, NSW Australia; 14 The Brain Cancer Group, North Shore Private Hospital, St Leonards, NSW Australia; 15 Department of Medical Oncology, Sir Charles Gairdner Hospital, Nedlands, WA Australia; 16 Medical School, University of Western Australia, Nedlands, WA Australia

**Keywords:** High grade glioma, Caregivers, Carer preparedness, Carer distress, Randomised controlled trial, Nurse-led intervention

## Abstract

**Background:**

High-grade glioma (HGG) is a rapidly progressing and debilitating disease. Family carers take on multiple responsibilities and experience high levels of distress. We aimed to deliver a nurse-led intervention (Care-IS) to carers to improve their preparedness to care and reduce distress.

**Methods:**

We conducted a randomised controlled trial (ACTRN:12612001147875). Carers of HGG patients were recruited during patients’ combined chemoradiation treatment. The complex intervention comprised four components: (1) initial telephone assessment of carer unmet needs; (2) tailored hard-copy resource folder; (3) home visit; and, (4) monthly telephone support for up to 12 months. Primary outcomes included preparedness for caregiving and distress at 2, 4, 6 and 12 months. Intervention effects were estimated using linear mixed models which included a time by group interaction. Secondary outcomes included anxiety, depression, quality of life, carer competence and strain.

**Results:**

We randomised 188 carers (n = 98 intervention, n = 90 control). The intervention group reported significantly higher preparedness for caregiving at 4 months (model β = 2.85, 95% CI 0.76–4.93) and all follow-up timepoints including 12 months (model β = 4.35, 95% CI 2.08–6.62), compared to the control group. However, there was no difference between groups in carer distress or any secondary outcomes.

**Conclusions:**

This intervention was effective in improving carer preparedness. However, carer distress was not reduced, potentially due to the debilitating/progressive nature of HGG and ongoing caring responsibilities. Future research must explore whether carer interventions can improve carer adjustment, self-efficacy and coping and how we support carers after bereavement. Additionally, research is needed to determine how to implement carer support into practice.

**Supplementary Information:**

The online version contains supplementary material available at 10.1007/s11060-023-04239-0.

## Introduction

Brain cancer in Australia is the sixth leading cause of cancer burden with a 22% five-year relative survival rate [1]. Annually in Australia, more than 2000 new cases of brain and other central nervous system cancers are diagnosed and 1477 Australians die from this disease [2]. Worldwide, approximately 300,000 people are diagnosed with brain and other central nervous system cancers annually [3]. In the United States the age adjusted incidence rate for malignant brain tumours is estimated at 7% per 100,000 people and the incidence rate for glioblastoma 3.21 per 100,000 people [4]. The mortality rate of cancer of the nervous system is estimated at approximately 3.4/100,000 globally [5].

High-grade glioma (HGG) includes grade III anaplastic astrocytomas, oligodendrogliomas and glioblastoma (grade IV). Patients diagnosed with astrocytomas and oligodendrogliomas, IDH mutant, grade 3, may live greater than 5 years [6], which can be in stark contrast to the shorter course of glioblastoma, IDH wildtype which can have a prognosis of 15 months [7, 8].

Despite different prognoses, all HGG diagnoses are debilitating. Adults diagnosed with HGG experience functional and neurological deficits, cognitive decline, and behavioural and personality changes [9]. Neurological symptoms include aphasias, ataxia, immobility, and cognitive changes, with impacts ranging from causing minimal symptoms to the patient being fully care-dependent [10], which is highly distressing for patients and their families. Our previous work highlighted the changing needs of patients following a HGG diagnosis and described four different trajectories of distress in patients, which could move from low to high, high to low, remain high or remain low, with some predictors of pattern [11].

Due to the progressive nature of HGG, carers rapidly transition into a carer role soon after diagnosis to manage the patients’ cognitive, personality, and functional changes and help them maintain their independent activities of daily living [12]. Carers’ responsibilities may include: communication with health professionals on treatment decision-making; transportation; managing symptoms, treatment side-effects (e.g., seizures and mobility difficulties); and, medication administration [12]. Consequently, they experience increased levels of anxiety, distress, information needs, and reduced quality of life [10, 13–17]. Our previous studies with carers of patients diagnosed with HGG demonstrate carers have high levels of distress during combined chemoradiation and for the subsequent 6 months [15, 18]. Patients and their carers require timely access to support and evidence-based information to manage their disease and its devastating impacts [19].

Three systematic reviews have been conducted to explore supportive interventions available for “family members of seriously ill patients in hospital” [20], “effectiveness of psychoeducational interventions for carers of patients with cancer” [21] and “evidence-based interventions for carers of patients with cancer to reduce family carer strain and burden” [22] with all concluding there are very few studies on effectiveness, highlighting the need for high quality studies with longer follow-up periods to demonstrate the feasibility and effectiveness of interventions. Structured support for family carers of patients with a terminal illness can improve carer preparedness, competence, psychological well-being and reduce unmet needs [23]. Spetz et al.’s [24] action research study found that providing patients and their family members (n = 16 dyads) with access to a specialist nurse was beneficial because family members felt less stressed and more supported because they formed a close relationship with the nurse and were able to share their concerns throughout the patients’ cancer journey.

Few randomised controlled trials (RCTs) or smaller single-arm studies have been conducted to improve support provided to carers of patients with brain cancer [25–27]. Philip and colleague’s [28] single-arm I-CoPE pilot intervention study (supportive care intervention delivered by cancer care coordinator consisting of (1) staged information, (2) regular screening for needs, (3) communication and coordination, and (4) family carer engagement demonstrated carers of patients with newly diagnosed HGG (n = 31) had fewer information and unmet supportive care needs and greater preparedness to care at 12 weeks compared to baseline. Boele et al. [29] pilot RCT provided patient-carer dyads (n = 56) with six psychologist-led sessions comprising education on disease-specific symptoms and cognitive behavioural therapy to increase coping with caring. These structured sessions helped carers maintain their mental functioning and improved their sense of mastery. Reblin et al. [30] used a two-group randomised design to test the effect of providing an electronic Social Network Assessment program to carers of brain cancer patients, showing carers in the intervention group (Total = 40, 30 intervention, 10 control) were significantly less depressed; however, anxiety remained stable. Building on this study, Reblin et al. [30] are currently conducting a randomised wait-list controlled trial testing whether an 8-week intervention for carers of patients with brain tumours consisting of an electronic Social Network Assessment program and caregiver navigator support including weekly phone sessions improves carer well-being.

Leveraging this work, our Care-IS RCT aimed to deliver a nurse-led intervention to carers of patients with HGG to improve their preparedness to care and reduce distress. We hypothesised carers who receive the intervention will feel more prepared for caring and experience less psychological distress as the patient’s disease progresses. We also proposed that improving carer preparedness and reducing their distress would improve patient outcomes including patient hospital admissions and length of stay. Secondary outcomes included anxiety, depression, quality of life, carer competence, and strain. We also collected data on carer unmet needs and healthcare resource utilization and costs which will be reported elsewhere. Here we report the primary endpoints: carer preparedness and carer distress and the following secondary outcomes: anxiety, depression, quality of life, carer competence and strain.

## Methods

A multistate Phase III RCT was conducted with eight Australian sites, three in Perth, WA and five in Sydney, NSW. CONSORT guidelines guided recruitment, monitoring of response rates, participant withdrawal and reporting.

Ethics approval was gained from participating sites (NSW: HREC 16/105; SJOG: 671; SCGH: 2013-172; Curtin University: HR 17/2013). Trial registration number: Australian and New Zealand Clinical Trials Registration (ACTRN) 12612001147875. We have previously published the protocol for the RCT [31] and reported on the feasibility testing and refinement of the Care-IS intervention [32].

### Participants

Carers of HGG patients were recruited during patients’ combined chemoradiation treatment. Eligibility criteria included: being a carer of a patient with HGG currently undergoing active treatment (chemotherapy, radiation therapy or combined chemoradiation); within 2 months of initial diagnosis; age 18 years and above; and, sufficient understanding of verbal and written English.

### Recruitment

Screening for eligibility was carried out by the medical oncologists, radiation oncologist, neuro- surgeons, or neuro-oncology cancer nurse coordinator, at the start of treatment for HGG. As the carer was often present for the patients’ medical appointments, clinicians discussed the trial with both the patients and their carers and referred interested carers to the study team. After potential participants were identified the research assistants invited the carer and patients to participate and provided information about the trial.

### Randomisation

Participants were randomised when they had completed the informed consent and baseline questionnaire. Participants were stratified by participating site and the patient’s Eastern Cooperative Oncology Group (ECOG) score (0–1 or ≥ 2) to ensure an even distribution between arms was achieved.

Block randomisation to treatment arm within each stratification was carried out using a computer-generated randomised table. Allocation to the treatment arms was centralised with the principal investigator at Curtin University allocating participants to trial arms after informed consent and baseline data collection. This method ensured the nurses/research assistants who had initial contact with the patients/carers and responsible for data collection were not involved in allocating participants to trial arms. Individual participants were informed of their allocated treatment arm; blinding was not possible due to the nature of the intervention.

### Intervention

The Care-IS intervention was developed by our multidisciplinary team (including a variety of stakeholders and consumer representatives) using the UK Medical Research Council Framework for developing and evaluating complex interventions [33]. Hudson’s [34] conceptual model for guiding supportive interventions for family carers of people receiving palliative care informed development of the intervention. This model focused on addressing the following areas for carers of patients receiving palliative care: preparedness to care, sense of control, competence, self-efficacy, anxiety, depression and distress, social support, information, a sense of reward, meaningfulness, positive emotions, optimism, respite and relationship with the patient. Intervention development has been further described in our protocol [31] and feasibility study [32] publications.

The Care-IS complex intervention comprised four components: (1) initial telephone assessment of carer unmet needs; (2) tailoring of a personalised resource folder; (3) home visit; (4) ongoing monthly telephone support for up to 12 months. All components of the intervention were documented and standardised in an evidence-based study manual which also contained resources for the intervention nurses to use (e.g., risk of falling; managing in an emergency). The following sections were included in the resource folder: Dealing with the diagnosis; caring for yourself; practical matters; communicating with friends or relatives; communicating with health providers; dealing with treatment, understanding physical symptoms and side effects; understanding mental and behavioural changes; lifestyle choices and complementary therapies; fertility and sexuality; and end of treatment. Carers received different parts of each section depending on the patients’ disease progression and the nurses’ assessment of the carers’ unmet needs.

During the telephone assessments the intervention nurses used the Palliative Care Needs Assessment Tool (PC-NAT) [35], the Integrated Palliative Outcome Scale (IPOS-5) [36], a nurse specific assessment developed for this trial and recorded details about the patient including their ECOG score. When nurses identified an unmet need they provided carers with recommendations and/or referrals to additional support services. Intervention nurses kept a detailed record of length of calls, problems or challenges identified, recommendations and referrals made. For the duration of the study three trained nurses provided the intervention. All three nurses were experienced oncology and palliative care nurses who received training focused on neuro-oncology, state specific services, the intervention and its delivery and communication skills. The three nurses reviewed each other’s intervention delivery, provided each other feedback and had regular debriefing sessions with each other and members of the investigator team. Where possible the same intervention nurse who provided the home visit provided the ongoing telephone follow-up for the duration of the carers’ participation in the study. This enabled the nurses to develop rapport, gain the carers’ trust and provide continuity of care.

### Usual care

The control arm received ‘usual care’ which included care provided at the site they were receiving treatment. This varied between sites, but consisted of the patients having access to a cancer care coordinator and/or oncology nursing staff and referral to palliative care. Full standard medical care and supportive management as clinically indicated occurred for patients in both arms of the trial. We have previously published a manuscript describing what usual care consists of in Australia and the differences that exist between sites [37].

### Measures

Primary outcomes included: carer preparedness measured using the Preparedness for Caregiving Scale [38]; and, carer distress measured using the Distress Thermometer [39] administered at baseline, 2, 4, 6, and 12 months. The preparedness for caregiving scale consists of 8 items where participants are asked to identify how well prepared they feel on a scale from not at all prepared (0) to very well prepared (4). The maximum score is 32 with higher scores indicating the carer is better prepared. The Distress Thermometer is a single item where participants indicate how distressed they have been in the last week from no distress (0) to extreme distress (10). Higher scores indicate that the carer is more distressed. The reliability of these instruments is discussed in our protocol [31].

Secondary outcomes included carer anxiety and depression measured using the Hospital Anxiety and Depression Scale (HADS) [40], carer quality of life measured by the Caregiver Quality of Life Index—Cancer [41], carer competence measured by the Carer Competence Scale [42] and carer strain measured by the Modified Caregiver Strain Index [43]. These measures have been described in further detail in our protocol [31]. All outcomes reported in this manuscript pertain to the carer except the ECOG performance status [44]. The patient ECOG status was collected for each time point and date of death was also collected for participants who died prior to data analysis.

### Sample size and power calculations

The sample size was calculated based on two co-primary end points: carer preparedness and carer distress at 4 months after randomisation. Using a 5% significance level, two-tailed testing of differences between two independent groups (usual care and intervention), a sample of 64 patients per group (128 total), has 80% power to detect group differences of 0.5 SDs (moderate effect size; considered clinically significant) for carer preparedness [45]. This sample size is based on means of 20 and 22.5 (SD = 5, range 0–32) for carer preparedness [46]. Having previously used the DT with carers [18], we hypothesised that we would reduce the number of carers with high levels of distress from the expected 33% in the control group to 13% (20% difference) in the intervention group at 4 months post-baseline. To detect this difference between groups (with 5% significance and two-tailed testing), we needed a sample size of 78 carers per arm (156 in total). Thus, a sample size of 156 at post-intervention testing would be sufficient for both primary end points.

### Data analysis

Descriptive statistics were reported as counts and percentages for categorical variables and means and standard deviations for continuous variables. Logistic regressions were performed to examine drop out at each time point. Caregivers who participated were dummy coded as 1 (retention) and those who dropped out were coded as 0 (drop out). Baseline characteristics were then entered as predictors of drop-out at each time-point. Results indicated unmarried, divorced or widowed caregivers were more likely to dropout at 2 months, and those who had reduced their working hours or stopped working were more likely to drop out at 4, 6 and 12 months (see Supplementary Table B). As a result these variables were adjusted for in the main analysis.

Linear mixed models (PROC Mixed procedure), with an unstructured correlation matrix, were used to estimate between-group differences in the primary and secondary outcomes at each time point and to determine the predicted least-square (LS) mean values. Each model included group (intervention or control) as a fixed effect, and time as both a fixed and random effect. Each model was adjusted for marital status and change in employment status (fitted as fixed effects), and also included a time by group interaction.

The effect of missing data was investigated as part of a sensitivity analysis using multiple imputation under the missing not at random (MNAR) assumption by searching for a tipping point that reverses the primary outcome conclusion [47]. Fixed values were added to randomly generated imputed values to investigate the impact on the primary outcome at the 4-month time point. Twenty-five data sets were created as described above.

All analyses were performed using Statistical Analysis Software (SAS/STAT) (version 9.4; SAS Institute, Cary, NC).

## Results

**Fig. 1:**
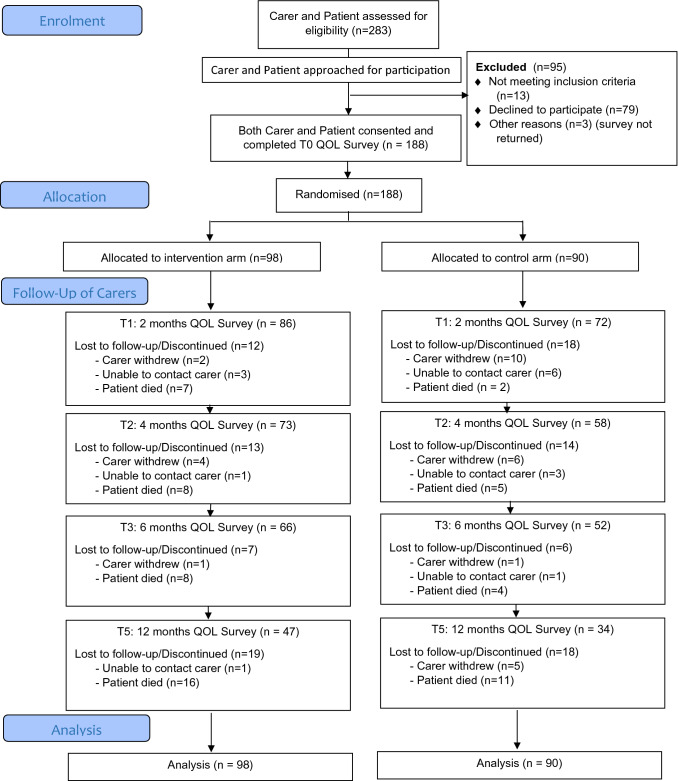
CONSORT flow diagram

One hundred and eighty-eight participants were randomly allocated across arms (n = 98 intervention, n = 90 control) (Refer Fig. 1). All participants were caring for a patient diagnosed with Grade III or Grade IV High Grade Glioma.

Baseline characteristics of the caregiver participants and the patients they cared for are displayed in Table 1. The majority of participants in both groups were female, had been caregiving for 1–3 months at the time of recruitment and were a spouse or partner of a patient with HGG. Average time from recruitment to patient death did not differ significantly between groups (M_intevention_ = 14.05 months, SD = 10.3; M_control_ = 14.11 months, SD = 8.12).

**Table 1:** Characteristics of Caregivers at Baseline (total N = 188)

	Intervention group N = 98 N (%)	Control group N = 90 N (%)
Patient characteristics		
Age (M,SD)	60.48 (11.1)	59.59 (12.6)
Gender		
Male	66 (67)	69 (77)
Female	32 (33)	21 (23)
ECOG status		
0	59 (60)	53 (59)
1	27 (28)	26 (29)
2	10(10)	7 (8)
3	2 (2)	2 (2)
4	0	1 (1)
Caregiver characteristics		
Age (M, SD)	57.61 (11.0)	56.54 (12.2)
Gender		
Male	28 (29)	20 (22)
Female	70 (71)	70 (78)
Length of caregiving		
> 1 month	21 (21)	17 (19)
1–3 months	58 (59)	49 (54)
4–6 months	7 (7)	13 (14)
6–12 months	2 (2)	3 (3)
> 12 months	0	3 (3)
Relationship to patient		
Spouse/Partner	83 (85)	82 (91)
Other (e.g. parent, child)	15 (15)	8 (9)
Marital status		
Married/partner	89 (91)	84 (93)
Widowed	1 (1)	0
Divorced/separated	1 (1)	2 (2)
Never married	7 (7)	4 (4)
Place of birth		
Australia	57 (58)	56 (63)
New Zealand	5 (5)	4 (4)
Fiji	1 (1)	0
Europe	26 (26)	18 (20)
Asia	3 (3)	6 (7)
Africa	4 (4)	2 (2)
North America	1 (1)	3 (3)
South America	0	1 (1)
English spoken at home		
Yes	90 (92)	82 (91)
No	6 (6)	8 (9)
Number of children (0–6)		
0	14 (14)	11 (12)
1–2	52 (53)	49 (54)
3–4	26 (26)	25 (28)
> 4	4 (26)	5 (6)
Number of children at home (0–4)		
0	55 (56)	49 (54)
1–2	25 (25)	24 (27)
3–4	6 (6)	8 (9)
> 4	0 (0)	0 (0)
Education		
High school	31 (32)	19 (21)
Postsecondary education	66 (68)	70 (79)
Employment status prior to diagnosis		
Full-time employed	37 (38)	35 (39)
Part-time employed	14 (14)	15 (17)
Unemployed	2 (2)	4 (4)
Retired	34 (35)	20 (22)
Other	11 (11)	15 (16.7)
Employment status change		
Stayed the same	87 (89)	69 (78)
Reduced hours or stopped	10 (10.3)	20 (23)
Financial effect of diagnosis		
No or slight effect	70 (72)	54 (60.6)
Significant effect	24 (25)	32 (36)

**Fig. 2:**
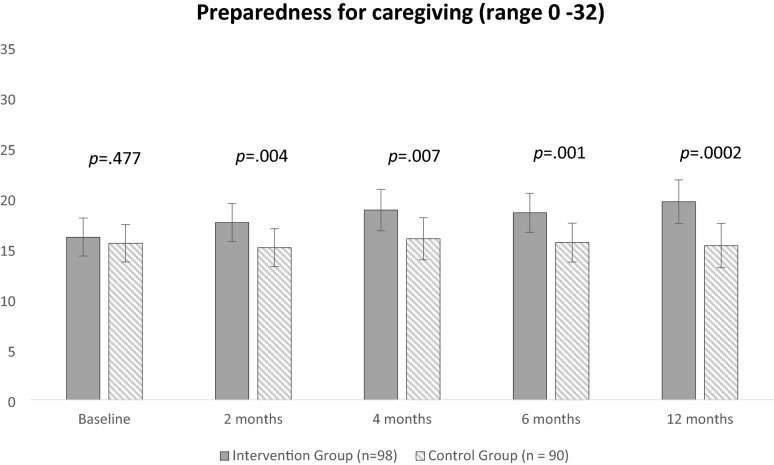
Least square mean values for preparedness for caregiving in the intervention (n = 98) versus control (n = 90) at baseline, 2, 4, 6, and 12 months

**Fig. 3:**
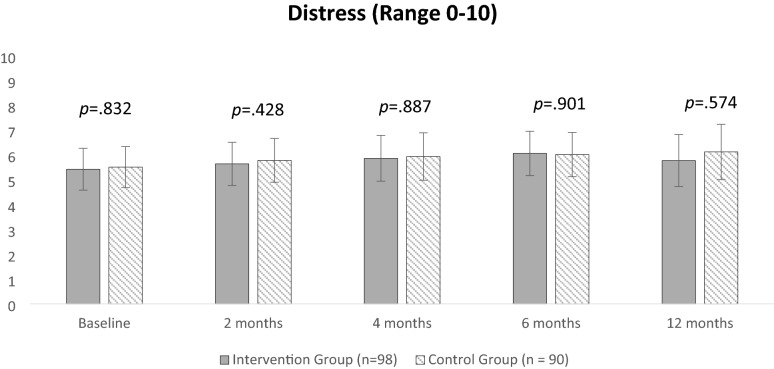
Least square mean values for distress in the intervention (n = 98) versus control (n = 90) at baseline, 2, 4, 6, and 12 months

Table 2 presents predicted LS mean values for primary and secondary outcomes for each group at each time point. The time by group interaction for the primary outcome of preparedness for caregiving was significant (*p* = 0.034) demonstrating that the two groups behave differently across all timepoints. After 4 months, intervention participants preparedness for caregiving was 2.85 points (*p* = 0.007) higher than the control group, and 4.35 points (*p* = 0.0002) higher at 12 months. The time by group interaction for the co-primary outcome of distress was non-significant (*p* = 0.972). The overall average difference in distress between groups was also non-significant (p = 0.775). Figures 2 and 3 provide a visual illustration of the predicted LS mean values for the co-primary endpoints in both groups over time.

**Table 2:** Comparison of caregiver outcomes in the intervention (n = 98) versus control (n = 90) at baseline, 2, 4, 6, and 12 months

Baseline Mean (SE)		2 months Mean (SE)		Mean difference	4 months Mean (SE)		Mean difference	6 months Mean (SE)		Mean difference	12 months Mean (SE)		Mean difference
IG	CG	IG	CG	Model β^a^ (95% CI)	IG	CG	Model β^b^ (95% CI)	IG	CG	Model β^c^ (95% CI)	IG	CG	Model β^d^ (95% CI)
Preparedness for caregiving (range 0–32)													
16.12 (0.96)	15.50 (0.95)	17.55 (0.97)	15.07 (0.96)	2.48 (0.76 to 4.19) *p* = 0.004**	18.80 (1.04)	15.95 (1.06)	2.85 (0.76 to 4.93) *p* = 0.007**	18.53 (0.99)	15.57 0.98)	2.95 (1.17 to 4.74) *p* = 0.001***	19.62 (1.10)	15.27 (1.11)	4.35 (2.08 to 6.62) ***p* = ** 0.0002***
Distress (range 0 to 10)													
5.42 (0.43)	5.51 (0.42)	5.64 (0.44)	5.78 (0.45)	− 0.14 (− 0.70 to 0.98) *p* = 0.742	5.86 (0.47)	5.93 (0.49)	− 0.07 (− 0.91 to 1.04) *p* = 0.887	6.06 (0.45)	6.01 (0.45)	0.05 (− 0.91 to 0.81) *p* = 0.901	5.77 (0.53)	6.12 (0.57)	0.35 (− 0.86 to 1.55) *p* =0 .574
Anxiety (range 0 to 21)													
10.21 (0.73)	10.52 (0.71)	10.21 (0.73)	10.19 (0.73)	0.02 (− 1.33 to 1.28) *p* = 0.970	10.38 (0.75)	10.90 (0.76)	− 0.52 (− 0.88 to 1.93) *p* = 0.467	10.54 (0.75)	10.62 (0.74)	− 0.12 (− 1.32 to 1.09) *p* = 0.850	10.90 (0.81)	11.10 (0.82)	− 0.20 (− 1.45 to 1.85) *p* = 0.816
Depression (range 0 to 21)													
8.29 (0.69)	8.35 (0.68)	8.77 (0.66)	8.49 (0.67)	0.28 (− 1.45 to 0.87) *p* = 0.629	8.48 (0.71)	8.16 (0.72)	0.33 (− 1.69 to 1.04) *p* = 0.639	8.54 (0.68)	8.43 (0.67)	0.11 (− 1.32 to 1.09) *p* = 0.850	9.56 (0.74)	9.57 (0.76)	− 0.01 (− 1.50 to 1.53) *p* = 0.983
QOL (range 0 to 140)													
75.65 (3.85)	73.43 (3.72)	76.26 (3.91)	72.28 (3.80)	3.98 (− 10.57 to 2.61) *p* = 0.235	76.84 (4.10)	71.93 (4.13)	4.91 (− 12.59 to 2.76) *p* = 0.209	78.36 (4.07)	71.71 (4.01)	6.66 (− 13.95 to 0.63) *p* = 0.073	73.62 (4.40)	66.23 (4.35)	7.38 (− 16.20 to 1.43) *p* = 0.100
Caregiver Competence (range 0 to 12)													
8.01 (0.40)	7.97 (0.39)	7.98 (0.39)	8.20 (0.39)	− 0.22 (− 0.48 to 0.92) *p* = 0.535	8.58 (0.41)	8.31 (0.41)	0.27 (− 1.04 to 0.50) *p* = 0.489	8.15 (0.41)	7.79 (0.41)	0.35 (− 1.11 to 0.40) *p* = 0.358	8.48 (0.43)	7.94 (0.44)	0.54 (− 1.42 to 0.34) *p* = 0.229
Caregiver Strain (range 0 to 26)													
15. 96 (0.95)	16.87 (0.94)	15.65 (0.97)	16.99 (0.98)	− 1.34 (− 0.41 to 3.09) *p* = 0.133	15.56 (0.97)	15.98 (1.02)	− 0.42 (− 1.46 to 2.29) *p* = 0.662	16.34 (1.00)	17.39 (1.01)	− 1.05 (− 0.79 to 2.90) *p* = 0.264	17.07 (1.10)	17.67 (1.15)	− 0.60 (− 1.72 to 2.92) *p* = 0.612

*IG* intervention group, *CG* control group

**p ≤ 0.01; ***p ≤ 0.001

^a^Mean difference between intervention versus control group at 2 months

^b^Mean difference between intervention versus control group at 4 months

^c^Mean difference between intervention versus control group at 6 months

^d^Mean difference between intervention versus control group at 12 months

With regard to the secondary outcomes, the time by group interactions for anxiety (*p* = 0.92), depression (*p* = 0.95), quality of life (*p* = 0.63), caregiver competence (*p* = 0.35) and caregiver strain (*p* = 0.82) were not significant. The overall average differences between groups in anxiety (p = 0.74), depression (p = 0.73), quality of life (p = 0.19), caregiver competence (p = 0.83), and caregiver strain (p = 0.22) were also not significant.

Sensitivity analyses examining preparedness for caregiving at the 4-month time point indicated if the missing values in the intervention group differed by more than − 0.80 points compared to the missing values in the control group, the difference in preparedness for caregiving between groups would be non-significant.

## Discussion

The Care-IS intervention was effective in improving carer self-reported preparedness to care at 4 months and subsequent points until 12 months when data collection ceased. This work builds on Boele et al.’s [29] pilot RCT which demonstrated carers of patients with HGG improved their sense of mastery when provided with a psychologist-led intervention consisting of education on disease-specific symptoms and cognitive behavioural therapy to increase coping with the caring role.

We found, despite improvements in carer preparedness, for carers who remained in the RCT carer distress was not significantly different at follow-up time points compared to participants in the control group. Similarly, QoL was not significantly different between groups. We propose changes did not occur in the intervention group due to the debilitating and progressive nature of HGG and ongoing caring responsibilities. This finding contrasts with Thakur et al.’s [48] small RCT (n = 80) which demonstrated a reduction in distress in carers of patients with intercranial tumours (majority had benign tumours) at one month follow-up. Of note, the follow-up timeframes for our trial compared to Thakur et al. may play a role, it may be possible to reduce distress over 4-weeks and in the context of benign disease, where the patients’ status is relatively stable compared to our population of HGG patients whose health is expected to deteriorate over 12-months. These findings emphasise the importance of longer-term follow-up of carers of HGG patients, including into the bereavement period to adequately assess the long-term impact of interventions which may include shorter bereavement period and/or reduced rate of complicated grief.

While we have demonstrated we can improve carers’ preparedness to care, reducing carers distress is challenging due to clinical context and declining trajectory of HGG patients. Coping with ongoing deterioration in health status of patients will present consistent new challenges and feelings of loss in carers, which this complex intervention was unable to mitigate in comparison to the control arm. Hudson et al.’s [49] RCT demonstrated that a psychological intervention for carers of patients referred for palliative care (n = 298) improved carer preparedness and competence in the short-term between referral to a palliative care service and one week after receiving the intervention. However, the carer cohort in the Hudson et al. RCT is not comparable to the Care-IS cohort, who were all caring for a person with significant cognitive, behavioral and physical disabilities. Additionally, the Care-IS intervention focused on supporting carers for up to 12 months. Carer competence for carers of patients diagnosed with HGG would consistently be challenged by the progressive deterioration of the patient, and the new roles and skills they need to occupy to provide the escalating care required. These ongoing, evolving concerns are unlikely to be addressed by this intervention which was designed to make them feel more prepared, rather than reduce the caring burden. Further research is warranted to determine whether carer interventions can improve carer adjustment, self-efficacy and coping skills and how we support carers after bereavement.

Usual care for patients diagnosed with HGG and their carers varies across Australia [37], with inconsistent availability of specialist services, timing of referral to palliative care, and poorly integrated primary care and community-based services. These findings in usual care and our RCT in carers highlights the need for additional support for carers and that care needs to be more wholistic to address the distress they experience in the immediate and longer-term. Additionally Schenker et al. [50] concluded a higher dose intensity supportive care intervention may be beneficial in improving patient reported outcomes when they found no difference in scores for symptom burden, mood and anxiety in their cluster RCT of an oncology nurse-led palliative care intervention for advanced cancer patients (consisting of 3 monthly visits with nurse). Interventions such as TeleMAST [51], a telephone delivered psychotherapeutic intervention that can be delivered to patients diagnosed with a brain tumour, patient-carer dyads, families, or carers alone, may effectively address some of the distress experienced and allow greater engagement with other supportive interventions and services over time.

While our intervention involved provision of information, support, and referrals for carers and patients, we do not currently know whether this advice was actioned by individual carers. There is a need to map referrals made to services accessed to understand the advice carers were able to implement. There is a need to explore, perhaps using mixed methods designs, the barriers to carer implementation of advice and/or referrals to overcome these barriers to care.

### Limitations

Our RCT was limited by only including English speaking carers living in Australia. There was a substantial drop-out across both arms in the trial, highlighting the challenges of longitudinal research in this population. Exploration of missing data via sensitivity analyses indicated if participants in the intervention group who dropped out at the 4-month time point reported worse preparedness for caregiving by 0.80 points compared to those who dropped out of the control group, the identified difference in preparedness for caregiving between groups would be non-significant. However, we have no reason to believe those who dropped out of the intervention group would be different to those who dropped out of the control group. Another potential factor may be the usual care provided at the recruitment sites, most of which had dedicated specialist brain cancer nurses on-site within tertiary care settings. Conceivably, this care had already maximized any changes in quality of life, anxiety, depression or caregiver strain which were able to be improved through nursing support, with more intensive nursing intervention unable to achieve additional gains.

## Conclusion

This intervention was effective in improving carer self-reported preparedness. However, carer distress was not reduced, potentially due to the debilitating and progressive nature of HGG and their ongoing caring responsibilities as the disease progresses. Future research needs to explore the type and timing of support carers of people with HGG would find most helpful and co-design strategies to implement their preferred type of support into usual care. Understanding the longer-term patterns of distress, into the bereavement period, may provide important insights into the effectiveness of interventions such as ours.

## Supplementary Information

Below is the link to the electronic supplementary material.

Supplementary file1 (DOCX 21 kb)
