# Supplementary material for: Carer preparedness improved by providing a supportive educational intervention for carers of patients with high-grade glioma: RCT results
Source: J Neurooncol. 2023 Jan 19;161(3):501–13. doi: 10.1007/s11060-023-04239-0 (PMC9992082; doi:10.1007/s11060-023-04239-0)
Supplement: Supplementary file 1 — Supplementary file1 (DOCX 21 kb) [file 11060_2023_4239_MOESM1_ESM.docx]

| **Supplementary Table A.** Completion Rates for all outcome measures | Intervention group (N = 98) | Control group (N = 90) | Total |
| --- | --- | --- | --- |
| **Baseline** |  |  |  |
| DT | 98 | 90 | 188 (100) |
| GHQ-12 | 94 | 84 | 178 (95) |
| HADS Depression | 98 | 89 | 186 (99) |
| HADS Anxiety | 98 | 89 | 187 (99.5) |
| Caregiver QOL | 80 | 62 | 142 (75.5) |
| PCS | 98 | 85 | 182 (97) |
| Caregiver competence | 97 | 86 | 183 (97) |
| Caregiver strain index | 94 | 84 | 178 (95) |
| **8 weeks** |  |  |  |
| DT | 81 | 61 | 142 (75.5) |
| GHQ-12 | 77 | 62 | 139 (74) |
| HADS Depression | 81 | 64 | 145 (77) |
| HADS Anxiety | 81 | 64 | 145 (77) |
| Caregiver QOL | 62 | 51 | 113 (60) |
| PCS | 78 | 61 | 139 (74) |
| Caregiver competence | 78 | 64 | 142 (75) |
| Caregiver strain index | 78 | 61 | 139 (74) |
| **4 months** |  |  |  |
| DT | 67 | 47 | 114 (61) |
| GHQ-12 | 66 | 43 | 109 (58) |
| HADS Depression | 69 | 47 | 116 (62) |
| HADS Anxiety | 68 | 47 | 115 (61) |
| Caregiver QOL | 48 | 32 | 80 (57) |
| PCS | 64 | 44 | 108 (57) |
| Caregiver competence | 67 | 46 | 113 (60) |
| Caregiver strain index | 64 | 40 | 104 (55) |
| **6 months** |  |  |  |
| DT | 58 | 52 | 110 (58) |
| GHQ-12 | 56 | 51 | 107 (57) |
| HADS Depression | 60 | 52 | 112 (60) |
| HADS Anxiety | 60 | 52 | 112 (60) |
| Caregiver QOL | 42 | 36 | 78 (41) |
| PCS | 58 | 48 | 82 (44) |
| Caregiver competence | 59 | 50 | 109 (58) |
| Caregiver strain index | 57 | 47 | 104 (55) |
| **12 months** |  |  |  |
| DT | 47 | 33 | 80 (43) |
| GHQ-12 | 47 | 33 | 80 (43) |
| HADS Depression | 47 | 32 | 79 (42) |
| HADS Anxiety | 46 | 33 | 79 (42) |
| Caregiver QOL | 28 | 23 | 51 (27) |
| PCS | 44 | 34 | 78 (41) |
| Caregiver competence | 47 | 32 | 79 (42) |
| Caregiver strain index | 45 | 31 | 76 (40) |

|  | T1 drop-out | T2 drop-out | T3 drop-out | T5 drop-out |
| --- | --- | --- | --- | --- |
| Explanatory variables | OR (95% CI) | OR (95% CI) | OR (95% CI) | OR (95% CI) |
| Gender  Female  Male | 0.96 (0.36-2.54) Ref | 1.03 (0.45-2.35)  Ref | 1.18 (0.50-2.74) Ref | 1.27 (0.56-2.88) Ref |
| Age | 1.00 (0.96-1.05) | 1.00 (0.96-1.04) | 1.03 (0.98-1.07) | 1.02 (0.98-1.06) |
| Length of Caregiving | 0.91 (0.76-1.10) | 0.93 (0.84-1.04) | 0.90 (0.78-1.03) | 0.99 (0.92-1.06) |
| Marital Status  Married/partner  Unmarried/divorced/widowed | 0.13 (0.03-0.62)** Ref | 0.63 (0.14-2.78)  Ref | 0.25 (0.05-1.25) Ref | 0.23 (0.04-1.46) Ref |
| Number of children living at home | 1.20 (0.77-1.86) | 1.17 (0.79-1.73) | 1.19 (0.79-1.77) | 1.16 (0.77-1.75) |
| Education Level  Uni/postgraduate Other form of lower education | 0.55 (0.14-2.23) Ref | 0.51 (0.16-1.56)  Ref | 0.46 (0.14-1.50) Ref | 0.45 (0.16-1.27) Ref |
| Country of birth Australia Other | 0.63 (0.27-1.49) Ref | 0.74 (0.35-1.55)  Ref | 0.60 (0.28-1.26) Ref | 0.40 (0.18-0.87)* Ref |
| Change in employment status Reduced hours/stopped  Stayed the same | 2.67 (0.85-8.40) Ref | 3.90 (1.23-12.38)*  Ref | 4.44 (1.39-14.20)* Ref | 5.14 (1.35-19.66)* Ref |
| Financial effect of diagnosis Significant effect No or slight effect | 1.42 (0.55-3.64) Ref | 1.93 (0.84-4.44)  Ref | 1.52 (0.65-3.59) Ref | 0.61 (0.226-1.46) Ref |
| Model Goodness of Fit Test | χ^2^(9, n=188) = 15.15  p = 0.87 | χ^2^(9, n=188) = 15.24  p = 0.85 | χ^2^(9, n=188) = 17.94  p = 0.036 | χ^2^(9, n=188) = 19.08  p = 0.024 |

**Supplementary Table B. Logistic Regression: Drop-out at each time point predicted by caregiver characteristics**

* = p ≤ .05, ** = p ≤ .01
